# Preferences for working in rural clinics among trainee health professionals in Uganda: a discrete choice experiment

**DOI:** 10.1186/1472-6963-12-212

**Published:** 2012-07-23

**Authors:** Peter C Rockers, Wanda Jaskiewicz, Laura Wurts, Margaret E Kruk, George S Mgomella, Francis Ntalazi, Kate Tulenko

**Affiliations:** 1 Department of Global Health and Population, Harvard School of Public Health, Boston, MA, USA; 2 Capacity Plus, IntraHealth International Inc., Washington, DC, USA; 3 Department of Health Policy and Management, Mailman School of Public Health, Columbia University, New York, NY, USA; 4 African Population and Health Research Center, Nairobi, Kenya; 5 Ministry of Health Republic of Uganda, Kampala, Uganda

## Abstract

**Background:**

Health facilities require teams of health workers with complementary skills and responsibilities to efficiently provide quality care. In low-income countries, failure to attract and retain health workers in rural areas reduces population access to health services and undermines facility performance, resulting in poor health outcomes. It is important that governments consider health worker preferences in crafting policies to address attraction and retention in underserved areas.

**Methods:**

We investigated preferences for job characteristics among final year medical, nursing, pharmacy, and laboratory students at select universities in Uganda. Participants were administered a cadre-specific discrete choice experiment that elicited preferences for attributes of potential job postings they were likely to pursue after graduation. Job attributes included salary, facility quality, housing, length of commitment, manager support, training tuition, and dual practice opportunities. Mixed logit models were used to estimate stated preferences for these attributes.

**Results:**

Data were collected from 246 medical students, 132 nursing students, 50 pharmacy students and 57 laboratory students. For all student-groups, choice of job posting was strongly influenced by salary, facility quality and manager support, relative to other attributes. For medical and laboratory students, tuition support for future training was also important, while pharmacy students valued opportunities for dual practice.

**Conclusions:**

In Uganda, financial and non-financial incentives may be effective in attracting health workers to underserved areas. Our findings contribute to mounting evidence that salary is not the only important factor health workers consider when deciding where to work. Better quality facilities and supportive managers were important to all students. Similarities in preferences for these factors suggest that team-based, facility-level strategies for attracting health workers may be appropriate. Improving facility quality and training managers to be more supportive of facility staff may be particularly cost-effective, as investments are borne once while benefits accrue to a range of health workers at the facility.

## Background

Governments in low- and high-income countries alike are struggling to attract and retain health workers in underserved areas. The World Health Organization (WHO) estimates that 57 countries worldwide are facing a critical shortage of health workers [1]. Further, those health workers that are practicing are predominantly located in urban areas, exacerbating the problem in rural settings. Health worker shortages reduce access to essential health care services, including immunizations and skilled birth attendance, and ultimately have a detrimental impact on population health. Indeed, health worker density has been shown to be negatively associated with maternal mortality, infant mortality and under-five mortality [2].

In order to operate properly and provide high quality care, health centers and hospitals require teams of providers and administrators with an appropriate mix of complementary skills and responsibilities [3]. Essential health workers include: 1) managers, 2) physicians or non-physician clinicians, 3) nurses, 4) auxiliary health personnel, including aides and technicians and 5) administrators. Difficulties in attracting and retaining particular health worker cadres in underserved areas have important implications for the functioning of entire health care teams. Without proper planning, understaffing in one cadre may force workers in other cadres to take on responsibilities for which they may have insufficient training or experience. For example, in many low-income countries, skilled nursing shortages often require auxiliary personnel to assume clinical responsibilities that they are not trained for [4]. This suggests that human resources for health strategies aimed at improving access to and quality of care in underserved areas need to consider the full health care team.

Recently, WHO published guidelines to assist ministries of health in determining the most appropriate policies to address issues of health worker attraction and retention in underserved areas [5]. They identified 16 key interventions, including health worker education and training, government regulations, financial incentives and personal and professional support programs. However, few low- or middle-income countries can afford to implement a number of different combinations of retention interventions in a trial and error approach to finding the “right” one. Rather, countries must determine which intervention package would be most effective in their local context, given local needs and resource constraints. Making this determination requires sound, local evidence. Strategies that aim to attract and retain full teams of essential health workers may be most effective in achieving the provision of high quality care at health facilities in underserved areas.

One method for assessing the potential effectiveness of strategies for attracting and retaining health workers in underserved areas is the discrete choice experiment (DCE). In research on human resources for health, DCE is used to elicit information on preferences for attributes of various incentive packages from practicing health workers or students in training programs [6,7]. This information can then be used to better understand how health workers may respond to future financial and non-financial incentives to practice at health facilities in rural areas. The literature on this topic includes studies conducted in both developing and developed countries, highlighting the relevance of the problem to policymakers throughout the world. Indeed, in a recent review of DCE studies of health worker job preferences, four of nine included studies were conducted in developed countries [8]. A primary advantage of the DCE method is that it produces not only a ranking of health worker preferences for various potential strategies, but also permits quantification of the value of one attribute compared to another. Few published articles on DCE have assessed multiple health worker cadres within the health system. Rather, most published articles have focused on a single cadre [6,7,9].

Conducting DCEs in low- and middle-income countries can present challenges to researchers that aren’t encountered when conducting similar studies in more developed countries. Mangham and colleagues have noted that cultural and language differences between researchers and study participants may be particularly challenging. The authors suggest that using local primary data to inform the design of the DCE instrument, and then pretesting the instrument in the local setting, is particularly important in developing country settings [10]. Further, determining the policy relevance of potential DCE attributes in developing countries requires a thorough understanding of local institutions and the available policy space. Efforts are underway to develop resources to make the DCE method more easily implementable in low- and middle-income countries. In particular, WHO, the World Bank and USAID’s global health workforce strengthening project Capacity*Plus* have collaborated to develop a practitioner’s guide for conducting DCEs for HRH policy in these settings [11].

The Republic of Uganda is a low-income country located in East Africa. The Uganda Ministry of Health (MOH) is currently facing challenges attracting and retaining health workers at facilities in the country’s rural areas. Indeed, vacancy rates in many rural areas of the country are as high as 60% [12]. Nearly three-quarters of the country’s doctors and 80% of the country’s pharmacists operate in urban centers, suggesting that producing more health workers will not solve shortages in rural areas [13]. At the time of the study, medical officers, the country’s physician cadre, received a base salary of around 700,000 USh (~300 USD) per month, while nursing officers, the country’s highest general nursing cadre, received a base salary of around 450,000 USh (~191 USD) per month. Housing allowances for all cadres were included in these base salary figures [14]. Further, all health workers working in hardship areas, as defined by the MOH, were provided a hardship allowance equal to 30% of their base salary. Finally, the MOH operated a bonding program whereby some students in health worker training programs agreed to work in a rural area for a set number of years (usually 2 years) after graduation in exchange for tuition support (personal communication, April 23, 2012).

As a result of these challenges, there are substantial gaps in access to essential health services in rural areas in Uganda. Only 42% of obstetric deliveries are attended by a skilled provider and less than two-thirds of children with acute respiratory infections receive care at a health facility [15]. Uganda has a high maternal mortality ratio (430 deaths per 100,000 live births) and a high infant mortality rate (79 deaths per 1,000 live births) [15]. The Ministry of Health has committed to addressing gaps in access to health services by pursuing strategies to attract and retain essential health workers in underserved areas. To this end the Ministry of Health, with technical assistance from Capacity*Plus*, conducted a DCE to better inform the selection of appropriate recruitment and retention interventions based on health worker preferences.

In this study, we investigate preferences for job attributes among students training to join four health worker cadres in Uganda, identified by the Ministry as being essential to a well functioning health facility that can provide primary and secondary health care: 1) medical officers, 2) nursing officers, 3) pharmacists and 4) laboratory technicians. A DCE was conducted with final year students in health training programs for these cadres. Mixed logit models were used to analyze the data. Results are compared across cadres to determine similarities in preferences that may indicate particularly effective strategies for health worker recruitment strategies in the country.

## Methods

### Sampling

Data for this study were collected from students in the final year of training programs for four cadres of health workers: 1) medical officer, 2) nursing officer, 3) pharmacist and 4) laboratory technician. These four cadres have been identified by the Uganda Ministry of Health as the minimum-staffing requirement needed to effectively run a public level-IV health center in the country. Level-IV health centers in Uganda are sub-district facilities that serve on average a catchment population of 100,000 persons [16]. These facilities are responsible for a wide range of curative, preventive, promotive and rehabilitative health activities [13]. All level-IV health centers should be equipped to admit inpatients and conduct emergency surgeries as well as provide emergency obstetric care. Sample sizes were determined primarily by logistic constraints related to student availability. While exact recommendations vary, a minimum of between 20 and 50 respondents per experiment group (in this case, health worker cadre) is required to reliably estimate respondent preferences [17]. It was determined during the study-planning phase that this minimum could be achieved despite constraints.

#### Medical students

All medical students in the fifth and final year of training at Makerere University, Mbarara University of Science and Technology (MUST) and Gulu University were invited to participate in the study. The programs at these three universities are the only public medical officer training programs in Uganda. There is one private university in the country that trains medical officers, Kampala International University (KIU). KIU was not included in this study because less than 10% of students in the medical training program at the university are Ugandan nationals.

#### Nursing students

Nursing students in the third and final year of the nursing officer training program at the Makerere University affiliated Mulago School of Nursing and Midwifery and at MUST were invited to participate in the study. A random sample of nursing students was drawn at the Mulago School because the training program was too large to interview all students. Of 228 eligible nursing students at Mulago, 125 were randomly selected for inclusion in the study. All third year nursing students at MUST were invited to participate in the study. At the time of data collection, Gulu University did not have a nursing officer training program. There are additional nursing officer training programs in Uganda at various smaller schools, including the Lira School of Nursing and the Jinja School of Nursing and Midwifery. However, students from these programs were not included in the analysis due to logistical constraints. Based on estimates of the number of nursing officer graduates produced each year in Uganda, we approximate that around 50% of all students in nursing officer training programs in the country were enrolled at the two schools sampled for this study [18].

#### Pharmacy students

All pharmacy students in the fourth and final year of training at Makerere University and MUST were invited to participate in this study. These are the only two pharmacist training programs in Uganda.

#### Laboratory students

All students in the second and final year of the laboratory technician training program at the Jinja School of Medical Lab Technology were invited to participate in the study. There is also a laboratory technician training program at Makerere University. However, students enrolled in this program were not available for inclusion in the study as they were on holiday during the data collection period.

Sampled universities were located in both urban (e.g., Makerere University in Kampala, Jinja School of Medical Lab Technology) and rural (e.g., MUST and Gulu University) settings, providing the variation necessary to investigate whether ruralness of the training program influences health worker job preferences.

### Instrument and survey fielding

All respondents first completed a short questionnaire with information on demographics and prior school and work experiences. Then, respondents completed a cadre-specific DCE that elicited preferences for attributes of potential future health sector jobs to be pursued after graduation. DCE scenario attributes and levels were informed by four activities: 1) a review of the published literature on strategies to attract and retain health workers, 2) discussions with members of the Uganda Ministry of Health, 3) eight focus groups—two with each of the four training programs under investigation and 4) pretests.

Focus group discussions (FGDs) were held at Mbarara University of Science and Technology (MUST), Makerere University and the Affiliated Mulago School of Nursing and Midwifery. At each location FGDs were held with each of the four health worker student groups of interest, including medical, nursing, pharmacy and laboratory students. Five to eight students participated in each FGD. FGD participants were not yet in the final year of their respective training programs, and were therefore not eligible to participate in the DCE. Prior to data collection, DCE instruments were pretested during interviews with local health workers. The project manager conducted the pretest interviews and took detailed notes. The information gathered during pretesting was then used to further refine the study instrument. For additional information on DCE instrument design see Additional file 1: the Technical Appendix.

Preliminary work indicated that six attributes were sufficiently important to warrant inclusion in the final DCE instruments. Five of six attributes were the same for each of the four cadre-specific DCEs (Table1): salary, quality of the health facility infrastructure and equipment, housing, length of time committed to the job posting and manager support. Overlap in attributes across cadres was not planned *ex ante* by the research team, but rather resulted from the described preliminary work conducted within each cadre independently. Levels for the salary attribute differed for each of the four instruments. For each cadre, the lowest salary level included in the instrument represented the actual salary for that cadre’s workers at the time of the study. Then, the incremental salary increases for each cadre were determined to be reasonable and policy relevant according to the MOH and FGD participants. The manager support attribute was meant to capture the influence of a health worker’s manager on the day-to-day work experience. The attribute had two levels: “the facility manager is supportive and makes work easier” or “the facility manager is not supportive and makes work more difficult.” Because medical officers most often report to the district health officer, the medical student DCE substituted “district health officer” for “facility manager.” DCE scenarios for medical students and laboratory technician students included tuition for future schooling as the final attribute. Scenarios for nursing students included health facility staffing as the final attribute while scenarios for pharmacy students included opportunities for private (dual) practice as the final attribute. See Additional files 2, 3, 4, 5 for copies of the instruments.

**Table 1:** DCE attributes and levels for four health worker training programs in Uganda

	**Health worker training program**			
	**Medical**	**Nursing**	**Pharmacy**	**Laboratory**
*Attribute 1*	*Salary*	*Salary*	*Salary*	*Salary*
Level 1^1^	“700,000 USh per month”	“450,000 USh per month”	“800,000 USh per month”	“400,000 USh per month”
Level 2	“1,000,000 USh per month”	“550,000 USh per month”	“1,200,000 USh per month”	“500,000 USh per month”
Level 3	“1,500,000 USh per month”	“650,000 USh per month”	“1,600,000 USh per month”	“600,000 USh per month”
Level 4	“2,000,000 USh per month”	“750,000 USh per month”	“2,000,000 USh per month”	“700,000 USh per month”
*Attribute 2*	*Facility quality*	*Facility quality*	*Facility quality*	*Facility quality*
Level 1	“Basic (e.g. unreliable electricity, equipment and drugs and supplies not always available)”	“Basic (e.g. unreliable electricity, equipment and drugs and supplies not always available)”	“Basic (e.g. unreliable electricity, equipment and drugs and supplies not always available)”	“Basic (e.g. unreliable electricity, equipment and drugs and supplies not always available)”
Level 2	“Advanced (e.g. reliable electricity, equipment and drugs and supplies always available)”	“Advanced (e.g. reliable electricity, equipment and drugs and supplies always available)”	“Advanced (e.g. reliable electricity, equipment and drugs and supplies always available)”	“Advanced (e.g. reliable electricity, equipment and drugs and supplies always available)”
*Attribute 3*	*Housing*	*Housing*	*Housing*	*Housing*
Level 1	“No housing or allowance provided”	“No housing or allowance provided”	“No housing or allowance provided”	“No housing or allowance provided”
Level 2	“Housing allowance provided, enough to afford basic housing”	“Housing allowance provided, enough to afford basic housing”	“Housing allowance provided, enough to afford basic housing”	“Housing allowance provided, enough to afford basic housing”
Level 3	“Free basic housing provided”	“Free basic housing provided”	“Free basic housing provided”	“Free basic housing provided”
*Attribute 4*	*Length of commitment*	*Length of commitment*	*Length of commitment*	*Length of commitment*
Level 1	“You are committed to this position for 2 years”	“You are committed to this position for 2 years”	“You are committed to this position for 2 years”	“You are committed to this position for 2 years”
Level 2	“You are committed to this position for 5 years”	“You are committed to this position for 5 years”	“You are committed to this position for 5 years”	“You are committed to this position for 5 years”
*Attribute 5*	*Support from manager*	*Support from manager*	*Support from manager*	*Support from manager*
Level 1	“The district health officer is not supportive and makes work more difficult”	“The district health officer is not supportive and makes work more difficult”	“The district health officer is not supportive and makes work more difficult”	“The district health officer is not supportive and makes work more difficult”
Level 2	“The district health officer is supportive and makes work easier”	“The facility manager is supportive and makes work easier”	“The facility manager is supportive and makes work easier”	“The facility manager is supportive and makes work easier”
*Attribute 6*	*Future tuition*	*Facility staffing*	*Private opportunities*	*Future tuition*
Level 1	“The government will not provide any financial assistance for a study program after your commitment is over”	“You have extra responsibility because 50% of health worker positions are vacant”	“You are not allowed to own or operate any private retail pharmacies”	“The government will not provide any financial assistance for a study program after your commitment is over”
Level 2	“The government will pay your full tuition for a study program (e.g., specialty training) after your commitment is over”	“You have extra responsibility because 25% of health worker positions are vacant”	“You are allowed to own and operate 1 private retail pharmacy as long as you also work full time at the facility”	“The government will pay your full tuition for a study program (e.g., specialty training) after your commitment is over”
Level 3		“You don’t have extra responsibility because all health worker positions are filled”		

All salary figures presented in Ugandan Shillings (USh). 1 USD = 2,350 USh.

^1^Level 1 salary figures represent the current base salary for respective health worker cadres in Uganda.

DCE scenario alternatives were paired using an experimental design to optimize D-efficiency, maximize level balance and orthogonality, and minimize overlap among attribute levels using Sawtooth Software’s SSI Web package (Sawtooth Software Inc. 2007). Respondents were presented with 11 random tasks and 1 fixed choice task. Each choice task was comprised of two job scenarios. Respondents were asked, “Please tell us which of these job postings you prefer.” For additional information on DCE experimental design see Additional file 1: the Technical Appendix. Surveys were administered to respondents in groups of between 10 and 30 on computers in university labs using Sawtooth Software’s SSI Web CAPI program. Prior to beginning the DCE, all respondent groups were read a standard introductory script by project personnel. The purpose of the script was to acclimate respondents to the hypothetical nature of the DCE they were about to take. The introductory script directed respondents to consider all DCE job scenarios to be located in rural areas. In this manner, the responses elicited do not reflect preferences for rural versus urban settings, but rather they reflect preferences for job attributes conditional on a rural setting. Following the introductory script, respondents proceeded to complete the survey questionnaire and DCE at their own pace on the personal computer. On average, respondents took approximately 30 minutes to complete the survey. The study was conducted during June and July 2010.

All survey respondents provided written consent prior to participation. The Uganda Ministry of Health shared data with the authors for analysis and publication. Clearance to analyze the de-identified administrative data and publish results was obtained from the Institutional Review Board at Harvard University.

### Statistical analysis

Univariate statistics were calculated for demographic, education and work experience variables. Four main effects mixed logit models were fit to DCE data from the four student groups under investigation to estimate preferences for job attributes. Fixed choice task data were not included in primary analyses. All attribute variables were specified as having a random component except for salary, which was specified as fixed in all models. Further, all attribute variables were coded as dummy variables except for salary, which was specified as continuous in all models. An alternative-specific constant was included in all models. Willingness to pay estimates were calculated by dividing attribute coefficients by the salary coefficient for each model. Willingness to pay estimates convey in monetary terms respondents’ preferences for one level of an attribute as compared to the reference level. Finally, three interaction models were fit to data from each cadre to determine if university attended, gender or living in a rural area prior to joining the training program significantly affected preference estimates.

Several validity tests were conducted to determine the appropriateness of model specifications. A fixed (hold-out) choice task was included to test the predictive validity of the model. The fixed task was designed to test preference for facility-level attributes, i.e., quality of facility infrastructure and support from facility management, because focus group rankings and previous research suggested high preference for these attributes. The data were tested for dominant preferences by identifying respondents who always selected a job posting on the basis of one attribute irrespective of the levels of other attributes. Finally, the data were tested for non-satiation by identifying respondents who chose dominated scenarios, i.e., scenarios in which all attributes with a clear ordering of levels were worse than the competing scenario. For additional information on the statistical theory that underlies DCE methodology and statistical model specifications, see Additional file 1: the Technical Appendix. All mixed logit models were fit using Stata’s mixlogit command (StataCorp 2007), and were specified with 500 Halton draws.

## Results

Of 259 eligible medical students, data were collected from 246 respondents (95.0% response rate). Of 155 eligible nursing students, data were collected from 132 respondents (85.2% response rate). Of 51 eligible pharmacy students, data were collected from 50 respondents (98.0% response rate). Finally, of 67 eligible laboratory students, data were collected from 57 respondents (85.1% response rate).

### Demographics

Minorities of medical (34.2%), pharmacy (32.0%) and laboratory (15.8%) students were female, while a majority of nursing students (70.5%) was female (Table2). Further, majorities of medical (72.0%), pharmacy (74.0%) and laboratory (84.2%) respondents were between ages 18 and 24, while two-fifths (40.9%) of all nursing respondents were between ages 25 and 34. More than half of respondents in each student group had previously lived in a rural area for at least 1 year. On average, medical students had worked 0.2 years (SD 1.1) in the health sector before entering their training program, while pharmacy students had worked 0.1 years (SD 0.3) and pharmacy students had worked 0.7 years (SD 2.9). Respondents in the nursing program had worked in the health sector relatively longer before coming to school, with an average of 4.2 years (SD 6.8). Large majorities of respondents in each of the four programs indicated that they would consider working in a rural area after graduating.

**Table 2:** Descriptive statistics for final year students in health worker training programs in Uganda, 2010

	**Medical**		**Nursing**		**Pharmacy**		**Laboratory**	
	**(N = 246)**		**(N = 132)**		**(N = 50)**		**(N = 57)**	
	**n**	**(%)**	**n**	**(%)**	**n**	**(%)**	**n**	**(%)**
**Demographics**								
Female	84	(34.2)	93	(70.5)	16	(32.0)	9	(15.8)
Age								
18 – 24	177	(72.0)	55	(41.7)	38	(76.0)	48	(84.2)
25 – 34	66	(26.8)	54	(40.9)	11	(22.0)	7	(12.3)
35 – 44	3	(1.2)	18	(13.6)	1	(2.0)	2	(3.5)
45 – 54	0	(0.0)	4	(3.0)	0	(0.0)	0	(0.0)
55+	0	(0.0)	0	(0.0)	0	(0.0)	0	(0.0)
Currently married	10	(4.1)	45	(34.1)	1	(2.0)	2	(3.5)
Has children	17	(6.9)	53	(40.2)	4	(8.0)	3	(5.3)
Lived in rural area at least 1 year	133	(54.1)	114	(86.4)	31	(62.0)	52	(91.2)
**University program and work experience**								
Form of tuition payment								
Sponsored by government of Uganda	176	(71.5)	24	(18.2)	27	(54.0)	21	(36.8)
Sponsored by other group	8	(3.3)	5	(3.8)	5	(10.0)	13	(22.8)
Pay out-of-pocket	61	(24.8)	100	(75.8)	18	(36.0)	23	(40.4)
Years of work experience*, mean (SD)*	0.2	(1.1)	4.2	(6.8)	0.1	(0.3)	0.7	(2.9)
Worked in a rural area	9	(3.7)	47	(35.6)	0	(0.0)	5	(8.8)
Worked in rural area as part of school program	241	(98.0)	111	(84.1)	35	(70.0)	32	(56.1)
Working in rural area after graduation								
Very unlikely	34	(13.8)	6	(4.5)	6	(12.0)	3	(5.3)
Unlikely	81	(32.9)	28	(21.2)	23	(46.0)	10	(17.5)
Likely	103	(41.9)	73	(55.3)	19	(38.0)	34	(59.7)
Very likely	22	(8.9)	24	(18.2)	2	(4.0)	9	(15.8)
**Preferences for job posting**								
Would consider working in a rural area								
Yes	230	(93.5)	127	(96.2)	48	(96.0)	56	(98.3)
No	15	(6.1)	3	(2.3)	2	(4.0)	1	(1.8)

Note: values may not add to total N due to missing responses (refusal to answer).

### DCE analyses

Preference estimates for each of the four cadres are presented in Tables 3,4,5,6. Output from mixed logit models includes two parameter estimates: mean utility and standard deviation. Mean utility coefficients are interpreted as relative preference weights where larger values indicate greater utility and more preferred attributes. Standard deviation estimates reflect preference heterogeneity in the population, a possible indication of unmeasured factors influencing the strength and direction of preference [19]. In Table3,4,5,6 mean utility coefficients for the salary attribute are standardized to represent respondent preferences for $500,000 USh per month. This standard is somewhat arbitrary and is not meant to represent a meaningful salary level for any cadre. The mean utility coefficient for salary is assumed to be constant at all salary levels and can easily be rescaled. Mixed logit estimates cannot be compared meaningfully across cadres due to incongruent utile scaling. Willingness to pay estimates (Table 7) accord to a standard scale and allow for cross-cadre comparison.

**Table 3:** Results of a mixed logit model of DCE data from medical students in Uganda, 2010

	**Mean**	**(SE)**	**SD**	**(SE)**
**Attribute**				
Quality of facility: advanced	0.85	(0.09)***	0.87	(0.10)***
Housing:				
Allowance provided	0.63	(0.09)***	0.05	(0.19)
Housing provided	0.59	(0.09)***	0.03	(0.15)
Tuition for future schooling	1.34	(0.10)***	0.95	(0.10)***
Length of commitment 2 yrs (ref: 5 yrs.)	0.80	(0.09)***	0.80	(0.10)***
Manager is supportive	0.47	(0.07)***	0.40	(0.12)***
Salary (continuous in 500,000^1^ USh/mo.)	0.93	(0.05)***		
Alternative-specific constant	0.12	(0.06)*		
**Model diagnostics**				
Number of respondents	246			
Number of observations	5,412			
Log likelihood	−1,241.0			
Likelihood ratio χ^2^	130.0			

*p < 0.10, **p < 0.05, ***p < 0.01.

^1^1 USD = 2,350 USh.

**Table 4:** Results of a mixed logit model of DCE data from nursing students in Uganda, 2010

	**Mean**	**(SE)**	**SD**	**(SE)**
**Attribute**				
Quality of facility: advanced	1.08	(0.14)***	1.16	(0.15)***
Housing:				
Allowance provided	0.56	(0.11)***	0.18	(0.33)
Housing provided	0.58	(0.11)***	0.05	(0.26)
Staffing level at facility (ref: 50% understaffed)				
25% understaffed	0.19	(0.11)*	0.00	(0.18)
Fully staffed	0.46	(0.11)***	0.36	(0.23)
Length of commitment 2 yrs (ref: 5 yrs.)	0.02	(0.07)	0.03	(0.31)
Manager is supportive	0.83	(0.12)***	0.95	(0.13)***
Salary (continuous in 500,000^1^ USh/mo.)	3.22	(0.27)***		
Alternative-specific constant	0.05	(0.08)		
**Model diagnostics**				
Number of respondents	132			
Number of observations	2,904			
Log likelihood	-728.5			
Likelihood ratio χ^2^	114.0			

**Table 5:** Results of a mixed logit model of DCE data from pharmacy students in Uganda, 2010

	**Mean**	**(SE)**	**SD**	**(SE)**
**Attribute**				
Quality of facility: advanced	0.85	(0.23)***	0.85	(0.29)***
Housing:				
Allowance provided	0.87	(0.25)***	0.25	(0.57)
Housing provided	0.91	(0.24)***	0.04	(0.57)
Allowed to own and operate 1 private pharmacy	1.94	(0.35)***	1.52	(0.33)***
Length of commitment 2 yrs (ref: 5 yrs.)	0.72	(0.24)***	1.11	(0.26)***
Manager is supportive	0.73	(0.22)***	0.98	(0.27)***
Salary (continuous in 500,000^1^ USh/mo.)	1.02	(0.16)***		
Alternative-specific constant	0.02	(0.17)		
**Model diagnostics**				
Number of respondents	50			
Number of observations	1,100			
Log likelihood	-242.9			
Likelihood ratio χ^2^	50.1			

**Table 6:** Results of a mixed logit model of DCE data from laboratory science students in Uganda, 2010

	**Mean**	**(SE)**	**SD**	**(SE)**
**Attribute**				
Quality of facility: advanced	1.72	(0.35)***	1.93	(0.37)***
Housing:				
Allowance provided	0.91	(0.23)***	0.01	(0.31)
Housing provided	1.17	(0.26)***	0.57	(0.31)*
Tuition for future schooling	1.45	(0.24)***	0.92	(0.26)***
Length of commitment 2 yrs (ref: 5 yrs.)	0.11	(0.19)	0.91	(0.24)***
Manager is supportive	0.64	(0.17)***	0.55	(0.29)*
Salary (continuous in 500,000^1^ USh/mo.)	1.61	(0.26)***		
Alternative-specific constant	0.04	(0.15)		
**Model diagnostics**				
Number of respondents	57			
Number of observations	1,254			
Log likelihood	-274.6			
Likelihood ratio χ^2^	73.2			

*p < 0.10, **p < 0.05, ***p < 0.01.

^1^1 USD = 2,350 USh.

#### Medical students

Medical students had high preference for tuition for future schooling (β 1.34, P < 0.01) and good quality health facility infrastructure and equipment (β 0.85, P < 0.01) (Table3). Further, they preferred a commitment to the job posting of 2 years as compared to 5 years (β 0.80, P < 0.01) and manager support (β 0.47, P < 0.01). Medical students had similar preference for being provided housing (β 0.59, P < 0.01) as compared to being given housing allowance (β 0.63, P < 0.01). Finally, they preferred more salary to less (β 0.93, P < 0.01). Specifically, medical students valued a 500,000 USh increase in salary approximately as much as good quality health facility infrastructure and equipment (β 0.93 and β 0.85, respectively).

#### Nursing students

Nursing students had high preference for good quality health facility infrastructure and equipment (β 1.08, P < 0.01) and a supportive manager (β 0.83, P < 0.01) (Table4). They also preferred jobs at health facilities that were fully staffed (β 0.46, P < 0.01). Nursing students had similar preference for being provided housing (β 0.58, P < 0.01) as compared to being given housing allowance (β 0.56, P < 0.01). Nursing students had no preference for length of commitment in the presented job scenarios. Finally, nursing students preferred more salary to less (β 3.22, P < 0.01). Specifically, nursing students valued a 500,000 USh increase in salary approximately three times as much as good quality health facility infrastructure and equipment (β 3.22 and β 1.08, respectively).

#### Pharmacy students

Pharmacy students had high preference for the ability to own and operate a private pharmacy (β 1.94, P < 0.01) (Table5). Pharmacy students also preferred good quality health facility infrastructure and equipment (β 0.85, P < 0.01), a commitment to the job posting of 2 years as compared to 5 years (β 0.72, P < 0.01) and manager support (β 0.73, P < 0.01). Further, they had similar preference for being provided housing (β 0.91, P < 0.01) as compared to being given housing allowance (β 0.87, P < 0.01). Finally, pharmacy students preferred more salary to less (β 1.02, P < 0.01). Specifically, pharmacy students valued a 500,000 USh increase in salary slightly more than good quality health facility infrastructure and equipment (β 1.02 and β 0.85, respectively).

#### Laboratory students

Laboratory students had high preference for tuition for future schooling (β 1.45, P < 0.01) and good quality health facility infrastructure and equipment (β 1.72, P < 0.01) (Table6). They also preferred manager support (β 0.64, P < 0.01). Further, as with respondents in all other student groups, laboratory students had similar preference for being provided housing (β 1.17, P < 0.01) as compared to being given housing allowance (β 0.91, P < 0.01). Laboratory students had no preference for length of commitment for job scenarios. Finally, as with respondents in all other student groups, laboratory students preferred more salary to less (β 1.61, P < 0.01). Specifically, laboratory students valued a 500,000 USh increase in salary slightly less than good quality health facility infrastructure and equipment (β 1.61 and β 1.72, respectively).

### Demographic modifiers

Several interaction models were run to investigate the potential modifying effects of university attended, gender and past residence in a rural area on respondent preferences (data available from authors). Neither gender nor past residence in a rural area significantly modified preferences within any of the cadres. University attended was important only for medical students. Specifically, medical students in training at Makerere University in urban Kampala had significantly less preference for future study opportunities as compared to students at the rural universities of MUST and Gulu University.

### Willingness to pay

In this study, willingness to pay should be interpreted as willingness to give up salary in exchange for another attribute. Willingness to pay is presented in Table7. Respondents in all groups appear to be willing to trade salary for good quality health facility infrastructure and equipment and a supportive manager, though the magnitudes of salary they are willing to trade-off differ depending on cadre. Medical students and laboratory students had a high willingness to trade salary for full tuition support for a future training program. Finally, pharmacy students were willing to trade substantial salary increases for an opportunity to own and operate a private pharmacy in addition to working at a public health facility. There is some debate in the literature regarding the usefulness of exact magnitudes of willingness to pay estimates from DCE analysis [20,21]. It may be most appropriate to consider relative values when interpreting willingness to pay estimates.

**Table 7:** Point estimates of willingness to pay for job attributes among four essential health worker cadres in Uganda, 2010

	**Health worker training program**			
	**Medical**	**Nursing**	**Pharmacy**	**Laboratory**
Quality of facility: advanced	0.46	0.17	0.42	0.54
Housing: allowance	0.34	0.09	0.43	0.28
Housing: provided	0.32	0.09	0.44	0.36
Commitment: 2-years	0.43	0.01	0.35	0.03
Manager: supportive	0.25	0.13	0.36	0.20
Full tuition support for future training program	0.72	-	-	0.45
Allowed to own and operate 1 private pharmacy	-	-	0.95	-
Staffing: 25% understaffed	-	0.03	-	-
Staffing: Fully staffed	-	0.07	-	-

Note: All values presented in 1 million USh per month. 1 USD = 2,350 USh.

### Validity tests

We found that, among medical, pharmacy and laboratory students, there were no respondents with dominant preferences. Among nursing students, one respondent demonstrated a dominant preference for good quality health facility infrastructure and equipment. Results of a mixed logit model of DCE data from nursing students run without data from this student were not different from full sample results. Among medical, nursing, pharmacy and laboratory students, respectively, 5%, 8%, 3% and 6% chose a dominated scenario. These proportions of ‘irrational’ choices are well within the acceptable standard for DCE [22]. Potentially ‘irrational’ responses may reflect legitimate decision rules, and consistent with current practice these respondents were not removed from the dataset for analysis [23,24]. We examined the internal predictive validity of the mixed logit model by comparing respondents’ actual choice of clinic in the fixed choice task versus the choice predicted by the model for this task. For all cadres, the predicted choice was within at least six percentage points of actual respondent choice. For additional information on the results of validity tests, see Additional file 1: the Technical Appendix.

## Discussion

We present data on preferences for job attributes among medical, nursing, pharmacy and laboratory technology students in Uganda. These four cadres comprise an essential health worker team required to operate a health center in the country that can provide primary and secondary health care. Nearly all respondents indicated that they were willing to consider working in a rural area after graduation given adequate working conditions. However, a substantial proportion of respondents in each cadre indicated that it is unlikely that they will work in a rural area after graduation given the state of the available rural job postings at the time of the study.

In choosing health sector job postings, respondents in all student groups expressed strong preference for increasing salary levels. This finding underscores the importance of providing an appropriate salary to attract health workers to underserved areas. Previous research supports this finding [25-27]. There is, however, mounting evidence that salary is not the only factor that health workers consider when deciding where to work [6,7,28]. It seems plausible that there may be a threshold effect when it comes to the importance of salary to health workers. That is to say, there may be a minimum salary below which health workers discriminate between jobs based solely on salary and above which they begin to discriminate based on other, non-financial factors. Sensitivity analyses that assessed preferences for salary modeled as categorical rather than continuous demonstrated little evidence of a decreasing marginal preference for salary, evidence against a threshold in these data. Future research should focus on determining if such a threshold salary exists, and if so what factors may determine the threshold level in a given setting.

This study contributes to the mounting evidence that salary is not the only important factor that health workers consider when deciding where to work. Better quality health facilities and supportive facility managers were important to all student groups in determining where they would prefer to work. Kruk and colleagues found similarly that, among medical students in Ghana, improved facility equipment and supportive management were most strongly associated with job preference [6]. The similarities in preferences for these factors across student groups suggest that certain health facility-level strategies for attracting essential health workers to underserved areas, including strengthening facility infrastructure and improving facility management, may be particularly cost-effective, as investments in these strategies are borne once while the benefits accrue across all health workers attracted to work at a health facility. Using the willingness to pay values presented in Table7, we calculate that an intervention that improves a single health facility’s quality would, if we assume that the facility staffs one medical officer, two nursing officers, one laboratory technician and one pharmacist, be equivalent to increasing salaries by 1.76 million USh ($749 US) per month. These findings also highlight the importance of managers in ensuring a well functioning health system. Indeed, in addition to strengthening health facility operations and potentially improving health worker performance, investments in programs that strengthen health facility management may have additional benefits with regard to attracting health workers to underserved areas [29].

While housing support was important to all student groups, respondents did not express preference for provision of actual housing compared to receiving a housing allowance. This suggests that the Uganda Ministry of Health has flexibility in crafting the housing component of health worker benefit packages. For example, if the Ministry can construct and maintain housing structures for health workers at a cost that is less than the market price for housing, they should perhaps do so.

We explored some cadre-specific incentives. Medical and laboratory students both expressed strong preference for full tuition support for a future training program. This finding is consistent with previous research that has shown that tuition support strategies have been successful in attracting health workers to underserved areas [27]. However, implementing tuition support strategies often requires coordination between ministries of health, ministries of education and public and private universities. As is the case in Uganda, coordinating these organizations, each with disparate goals and agendas, can be difficult [13].

Pharmacy students placed high value on the opportunity to own and operate a private pharmacy in addition to working at a public health facility, referred to as dual practice or moonlighting. Assuming that the primary utility these students expect to derive from dual practice will take the form of increased income, we calculate, based on the results in Table7, that on average students expect a single private pharmacy to produce 1 million USh ($425 US) per month in income. Currently, the official policy of the Uganda Ministry of Health is to disallow dual practice by pharmacists in the country. These findings suggest that there may be an opportunity to reform dual practice regulations for pharmacists in the country in order to make postings in rural health facilities more attractive. However, policymakers should also consider the potential disadvantages of dual practice by pharmacists. Namely, providers engaging in dual practice may be prone to refer patients at public health facilities to their own private pharmacies in order to increase private financial gains [30]. This can result in inefficiency in the health system and contribute to higher out-of-pocket expenditures for families.

There were limitations to this analysis. First, as previously discussed, conducting a DCE in a resource limited setting such as Uganda presents unique challenges, including those that result from cultural differences between the research team and study participants. However, while the research presented here was supported by a team of non-Ugandans working for USAID’s Capacity*Plus* Project, Ugandan collaborators, including those from the Ministry of Health, provided extensive input at all stages of the study. Further, as described above, focus groups were conducted and pretesting was done to ensure that all attributes and levels included in the final DCE instruments were informed by local primary data. Finally, Ministry of Health collaborators ensured that all included job attributes were relevant to the policy process in Uganda. Second, our sample is limited to students still in training to become health workers. Further, student respondents had very limited experience living and working in rural areas. It is possible that health worker job preferences change as a result of experiences. There is evidence to suggest that important differences may exist between the preferences of students and active health workers [31,32]. The findings presented here should inform recruitment rather than retention strategies. Further, the final sample sizes for this study were determined in part by logistical considerations. However, as previously mentioned, the study achieves the recommended minimum sample size per cadre despite constraints. Third, the DCE administered in this study was unlabeled, i.e. the job postings described in the DCE scenarios were not identified as being in rural or urban settings. Including such labels would have provided a means to isolate the effect of ruralness on job preferences. However, we chose not to label the DCE scenarios because labels have been shown to distract respondents from job attributes and thus may diminish the reliability of estimates of attribute preferences.[33] We were primarily interested in understanding which attributes of rural job postings were most preferred by respondents. In the script delivered at the beginning of DCE administration, respondents were told to consider all job scenarios to be located in rural areas. However, we have no way to confirm that respondents fully committed to considering all DCE scenarios to be located in rural areas. Finally, the DCE results presented here constitute stated preferences and reflect responses to hypothetical scenarios. The results of a DCE such as this one have not, at this time, been demonstrated to accurately predict health worker behavior in making job market decisions. Future work on this point is important, and until the predictive power of DCE for HRH policy is determined, decision makers should consider DCE information as one of many inputs to the policymaking process.

## Conclusions

Information on health worker preferences for job attributes can help guide policymakers in deciding which strategies to pursue to attract essential health workers to underserved areas. Discrete choice experiments are a feasible policy tool that can be used in developing countries to elicit such preference information. Resources are increasingly being developed to guide practitioners in conducting DCEs for human resources for health (HRH) policy [10,17,34]. Indeed, the World Bank, the World Health Organization, and Capacity*Plus* have recently collaborated to develop a guide for conducting a DCE to inform HRH policy [11]. If the DCE method is to be used for policymaking, it’s important that investigators adhere to rigorous methodological standards to ensure that collected information is meaningful.

Staffing rural health facilities with full teams of essential health worker personnel is an important means to strengthen the health system in Uganda and other low-income countries. In Uganda, preference information suggests that both financial and non-financial incentives may be effective in attracting health workers to underserved areas. Medical, nursing, pharmacy and laboratory students all expressed high preference for increased salary. In addition, similarities across student groups in preferences for health facility-level strategies suggest potentially cost-effective interventions. Improving the quality of health facility infrastructure and training facility managers to be more supportive of provider personnel may be particularly effective. Our findings are consistent with recent studies of health worker preferences in other developing countries [8]. However, more work is required to determine the generalizability of DCE preference information across countries. The work presented in this article was conducted in collaboration with the Uganda Ministry of Health, and at the time of writing the Ministry was in the process of incorporating these findings, as one of several important sources of information, into a comprehensive HRH reform proposal.

## Competing interests

The authors declare that they have no competing interests.

## Authors’ contributions

PCR supervised study design and data collection and led the analysis and writing. WJ conceived the study and assisted with data collection and writing. LW assisted with the data collection and writing. MEK assisted with study design, analysis and writing. GSM assisted with data collection and writing. FN assisted with study design, data collection and writing. KT assisted with study design and writing. All authors read and approved the final manuscript.

## Pre-publication history

The pre-publication history for this paper can be accessed here:

http://www.biomedcentral.com/1472-6963/12/212/prepub

## Supplementary Material

Additional file 1 **Technical Appendix**[5-7,19,22-24,27,35-41]. Click here for file

Additional file 2 Medical student survey instrument. Click here for file

Additional file 3 Nursing student survey instrument. Click here for file

Additional file 4 Pharmacy student survey instrument. Click here for file

Additional file 5 Laboratory student survey instrument. Click here for file
