# Supplementary material for: Preferences for working in rural clinics among trainee health professionals in Uganda: a discrete choice experiment
Source: BMC Health Serv Res. 2012 Jul 23;12:212. doi: 10.1186/1472-6963-12-212 (PMC3444383; doi:10.1186/1472-6963-12-212)
Supplement: Additional file 1 — Technical Appendix[5-7,19,22-24,27,35-41]. [file 1472-6963-12-212-S1.doc]

**Technical Appendix**

*Instrument design and administration*

There are three general stages in the DCE design process: 1) identification of attributes, 2) assignment of levels and 3) selection of choice tasks [35].

Identification of attributes and levels

The selection of DCE scenario attributes and levels were informed by four activities: 1) a review of the published literature on strategies to attract and retain health workers, 2) discussions with members of the Uganda Ministry of Health, 3) focus groups and 4) pretests.

The recent WHO report, “Increasing access to health workers in remote and rural areas through improved retention,” outlines a set of financial and non-financial incentives that have previously been shown to be important to health workers when they are deciding where to work [5]. Based on this report, and other recent research related to health worker attraction and retention, an initial list of potentially important job attributes was compiled for health workers in Uganda [6,7,27]. Then, through meetings with key members of the Uganda Ministry of Health, those attributes that were most relevant in the Uganda context and that were most likely to reflect feasible policy options for the country were identified.

Next, eight focus group discussions (FGD) were held with students in health worker training programs in Uganda. Four focus groups were held at Mbarara University of Science and Technology (MUST) and four additional focus groups were held at Makerere University and the Affiliated Mulago School of Nursing and Midwifery. One FGD at each location was held with each of the four health worker student groups of interest, including medical, nursing, pharmacy and laboratory students. FGD participants were not yet in the final year of their respective training programs, and were therefore not eligible to participate in the DCE. All FGD participants gave written consent to participate. The project manager, trained in focus group methods, facilitated the FGDs. All FGDs were conducted in English and lasted approximately one hour.

Participants in focus groups were first asked general questions about their perceptions of health worker job postings in Uganda, and their previous experiences living in rural areas and working in the country’s health sector. Next, participants discussed factors that they considered to be important to them when they thought about where they may want to work after graduation. Participants were asked specific questions about job attributes that were previously identified as potentially being important, based on the literature review and meetings with the Uganda Ministry of Health, as described above. Then, participants were asked to individually rank these attributes according to what they believed to be most important to them. Finally, participants nominated a final set of attributes. For each attribute, participants were asked to identify levels that were realistic and appropriate in the local context. For example, when discussing salary, participants were asked to identify what they deemed to be a realistic and fair salary level for their future job posting.

Based on information collected during FGDs, six attributes were included in the final DCE instrument for each of the four student groups. Five of six attributes were the same for each of the four DCEs (Table 1): salary, quality of the health facility (with a focus on quality of equipment for laboratory technicians), housing, length of time committed to the job posting and manager support. Levels for these attributes, however, differed for each of the four instruments. DCE scenarios for medical students and laboratory technician students included tuition for future schooling as the final attribute. Scenarios for nursing students included health facility staffing as the final attribute, and scenarios for pharmacy students included opportunities for private (dual) practice as the final attribute.

Selection of scenarios

The number of possible job posting scenarios is calculated as a function of the number of attributes and levels. The medical, pharmacy and laboratory DCE had one attribute with four levels (salary), one attribute with three levels (housing) and four attributes with two levels. This design generated 192 (4^1^ x 3^1^ x 2^4^) possible scenarios. The nursing DCE had one attribute with four levels (salary), two attributes with three levels (housing and staffing) and three attributes with two levels. This designed generated 288 (4^1^ x 3^2^ x 2^3^) possible scenarios. For each DCE, the magnitude of possible scenarios is too many for any individual respondent to evaluate. As such, DCE scenario alternatives were paired using an experimental design to optimize D-efficiency, that is to maximize level balance and orthogonality, and to minimize overlap among attribute levels using Sawtooth Software’s SMRT package (Sawtooth Software Inc. 2007). Respondents were presented with eleven random tasks and one fixed choice task. Each choice task was comprised of two job scenarios and respondents were asked to select their preferred scenario.

A fixed (hold-out) choice task was included to test the predictive validity of the model. The fixed task was designed to test preference for facility-level attributes, i.e., quality of facility infrastructure and support from facility management, because focus group rankings and previous research suggested high preference for these attributes. In the fixed choice task, Job Posting A had poor facility quality and unsupportive management and good non-financial personal incentives (e.g., shorter time commitment, support for future tuition) whereas Job Posting B had good facility quality and supportive management and poor non-financial personal incentives (e.g., longer time commitment and no support for future tuition). Other attributes, including salary and housing, were held the same across scenarios.

Pilot testing

Prior to data collection, DCE instruments were pretested during interviews with local health workers. The project manager conducted pretest interviews and took detailed notes. The information gathered during pretesting was then used to further refine the study instrument. Important information included the perceived realism of the framing and design of the DCE, the appropriateness of included attributes and levels and any other difficulties encountered when taking the DCE.

Fielding

Data collection activities were conducted during August 2010. Surveys were administered on computers in university labs to groups of between ten and thirty students using Sawtooth Software’s SSI Web CAPI program. Prior to beginning the DCE, all groups of respondents were read a standard introductory script by project personnel. The purpose of the script was to acclimate respondents to the hypothetical nature of the DCE they were about to take. As is standard, respondents were asked to imagine making a real choice, take into account only the attributes described, and instructed that there were no right or wrong answers. Respondents then proceeded to complete the survey questionnaire and DCE at their own pace on their personal computer. On average, respondents took approximately thirty minutes to complete the survey. All respondents provided consent prior to participating in the study. All questions and DCE scenarios were presented in English.

*Analytic model*

DCE are based on random utility theory, which posits that an individual’s true latent but unobservable utility for a given alternative in a choice situation is comprised of a systematic component derived from preferences for known attributes of the scenarios under consideration, and a random component. Then, the probability of choosing a given alternative in a choice situation can be written as a standard logit distribution.

Recent work suggests that mixed logit models, however, may have advantages over standard logit models for analyses of DCE data [36]. As such, we employ mixed logit models for this study. Mixed logit models allow attribute coefficients to vary across respondents, accounting for preference heterogeneity and improving the realism of model assumptions. Second, mixed logit models adjust the standard errors of utility estimates to account for repeated choices by the same individual. Mixed logit models typically have better model fit than more standard methods, including multinomial and conditional logistic models [19]. Finally, mixed logit models are able to account for individual-specific factors such as demographic characteristics through the inclusion of interaction terms between a demographic variable and a DCE attribute [36].

*Statistical analysis*

We estimated four main effects mixed logit models of DCE data. Fixed choice task data were not included in primary analyses. All attribute variables were specified as having a random component except for salary, which was specified as fixed in all models. While random specifications of salary may improve model fit, a fixed coefficient ensures that the estimate of salary utility has the right sign and is preferred for calculation and interpretation of willingness to pay.[37] Further, all attribute variables were coded as dummy variables except for salary, which was specified as continuous in all models. An alternative-specific constant was included in all models. Several validity tests were conducted to determine the appropriateness of model specifications, including tests for predictive validity, dominant attributes and irrational respondents. Output from mixed logit models includes two parameter estimates: mean utility and standard deviation. Mean utility coefficients are interpreted as relative preference weights where larger values indicate greater utility and more preferred attributes. Standard deviation estimates reflect preference heterogeneity in the population, a possible indication of unmeasured factors influencing the strength and direction of preference [19]. All mixed logit models were fit using Stata’s mixlogit command, and were specified with 500 Halton draws (StataCorp 2007).

*Validity tests*

Dominant preferences

The data were tested for dominant preferences by identifying respondents who always selected a job posting on the basis of one attribute irrespective of the levels of other attributes. Dominant preferences represent non-compensatory behavior and violate random utility theory[38]. We found that, among medical, pharmacy and laboratory students there were no respondents with dominant preferences. Among nursing students, one respondent demonstrated a dominant preference for good quality health facility infrastructure and equipment. Results of a mixed logit model of DCE data from nursing students run without data from this student were not different from full sample results.

Non-satiation

We performed a test for non-satiation by identifying respondents who chose dominated scenarios, i.e., scenarios in which all attributes with a clear ordering of levels (salary, health facility quality, management, tuition support and opportunity for dual practice) were worse than the competing scenario[39]. For example, a dominated scenario for medical students would have lower salary, poor health facility quality, unsupportive management and no tuition support. Choice tasks with a dominated scenario were identified for each of the four student groups. Among medical students, 5% chose a dominated scenario. Among nursing students, 8% chose a dominated scenario. Among pharmacy students, 3% chose a dominated scenario. Among laboratory students, 6% chose a dominated scenario. These proportions of ‘irrational’ choices are well within the acceptable standard for DCE [22]. Potentially ‘irrational’ responses may reflect legitimate decision rules, and consistent with current practice these respondents were not removed from the dataset for analysis [23, 24].

Internal validity: predictive and convergent validity

We examined the internal predictive validity of the mixed logit model by comparing respondents’ actual choice of clinic in the fixed choice task (same clinic attributes shown to all respondents) versus the choice predicted by the model for this task. For medical students, the final model predicted that 66% of respondents would prefer the high quality facility with supportive management; 72% of medical students actually selected this scenario. For nursing students, the final model predicted that 74% of respondents would prefer the high quality facility with supportive management; 75% of nursing students actually selected this scenario. For pharmacy students, the final model predicted that 63% of respondents would prefer the high quality facility with supportive management; 62% of pharmacy students actually selected this scenario. For laboratory students, the final model predicted that 51% of respondents would prefer the high quality facility with supportive management; 47% of laboratory students actually selected this scenario. These findings suggest that the final model specifications used to analyzed DCE data were correct.

*Alternate model specifications*

Variable coding

We investigated alternative variable coding methods to determine the most appropriate model specifications. The salary variable was modeled alternatively as categorical, linear and quadratic. The linear specification was determined to be the best fit and was used for all final models. All dichotomous and categorical attribute variables were alternatively modeled with dummy and effects coding. Results from models with effects coding demonstrated no important change in the value of the coefficient for the constant, suggesting the constant was not affected by choice of coding technique. We thus elected to retain dummy coding as it is standard in the DCE literature.

Correlated attributes

We re-specified our model allowing for correlation between attributes, i.e., assuming non-independence of job posting characteristics. This is done by specifying a general covariance pattern across the coefficients within the mixlogit command in Stata [40]. This specification did not materially change coefficient estimates.

*Simulated uptake*

We simulated potential job posting uptake given specific health system reforms, using preferences estimated by the main effects mixed logit models [41]. Stata’s MIXLPRED command was used for all simulations. MIXLPRED operates by applying individual-level coefficient estimates derived from the mixed logit model to predict choice of a job posting given competing posting scenarios.

In the simulations, we compared job posting with improved attributes (e.g., as a result of reform efforts) to a baseline job posting with attributes found in rural Uganda today. The results of select simulations are presented in the table below.

|  | **Improved job posting^1^** | | | |
| --- | --- | --- | --- | --- |
| **Simulated scenario** | **Medical** | **Nursing** | **Pharmacy** | **Laboratory** |
| Current situation | 50% | 50% | 50% | 50% |
| 30% increase in salary | 60% | 70% | 62% | 60% |
| 50% increase in salary | 66% | 81% | 69% | 66% |
| Advanced facility quality | 68% | 70% | 68% | 75% |
| Supportive facility manager | 61% | 67% | 65% | 64% |
| Full tuition support | 76% | - | - | 78% |
| Full staffing | - | 61% | - | - |
| Dual practice allowed | - | - | 80% | - |

^1^As compared to a baseline job posting defined as: current salary (medical: 700,000 USh; nursing: 450,000 USh; pharmacy: 800,000 USh; laboratory: 400,000 USh); basic facility quality; housing allowance provided; length of commitment 5 years; facility manager not supportive; no future tuition support (for medical and laboratory); 50% understaffed (for nurses); no dual practice (for pharmacy).

These results confirm the importance of health facility quality and manager support in attracting and retaining health workers to job postings in underserved areas.
