# Supplementary material for: Preferences for working in rural clinics among trainee health professionals in Uganda: a discrete choice experiment
Source: BMC Health Serv Res. 2012 Jul 23;12:212. doi: 10.1186/1472-6963-12-212 (PMC3444383; doi:10.1186/1472-6963-12-212)
Supplement: Additional file 4 — Pharmacy student survey instrument. [file 1472-6963-12-212-S4.pdf]

Start

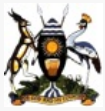

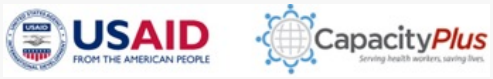

Determining priority retention packages for costing to increase attraction and retention in rural and remote areas.

Thank you for coming today. We are working with the Uganda Ministry of Health to learn about the incentives and motivating factors that drive health care providers to move to under-served areas. As current pharmacy students looking towards the future, we are interested in the way you consider different incentives and opportunities to practice in diverse settings. This survey should take approximately 20-30 minutes. Your participation will help us quantify the attractive power of incentives and characteristics to motivate health workers to accept postings in rural areas.

Please continue to the next page to read an electronic consent form.

Next

Consent

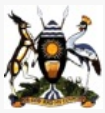

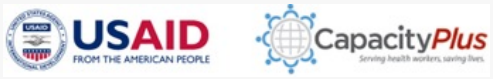

Determining priority retention packages for costing to increase attraction and retention in rural and remote areas.

Informed Consent

Introduction:
You are being asked to take part in this activity by the Ministry of Health to examine the factors affecting health worker motivation for rural practice in Uganda in order to cost priority retention packages. In order to ensure that you are informed about this activity, we are asking you to read this consent form. You will also be asked to sign it. We will give you a copy of this form. Please ask us to explain anything you do not understand.

Purpose of the Activity:
This activity is being conducted by the Ministry of Health in collaboration with CapacityPlus a project working to strengthen human resources for health systems around the world. We will gather information mainly on preferences for job attributes for postings in rural areas. Basic demographic information, including gender and age, will also be collected.

Your Part in the Activity:
If you agree to participate in the survey you will be asked to complete a survey questionnaire that takes approximately 30 minutes. About 300 people will take part in the survey in Uganda.

If You Decide not to Participate in the Study:
Your participation in the study is voluntary, and there is no penalty for refusing to take part.

Confidentiality:
The information you provide will be confidential. We will not put your name on the questionnaire form on which your responses will be recorded. If we publish the results of the activity your name will not be in it.

Benefits:
There is no financial compensation or other personal benefits from participating in this activity. However, your participation and/or answers to the questions may provide insights into the best strategies for designing future incentives packages to help attract and retain health professionals in rural areas.

Risks or Discomfort:
There are no known risks to you resulting from your participation in the activity. If you experience any personal discomfort during the interview you may, as stated above, stop the interview at any time or refuse to answer any questions.

Contact Person for Questions:
If you have any questions about this activity you may contact Norah Nandudu at 0782-331-865.

Consent to Participate:
I have read the foregoing information. I have had the opportunity to ask questions about it and any questions I have asked have been answered to my satisfaction. By clicking YES below, I certify that I am at least 18 years old and consent voluntarily to participate as a subject in this study. I understand that I have the right to withdraw from the study at any time without in any way it affecting my academic standing.

- YES (continue to survey)
- NO (terminate survey)

Next

0% 100%

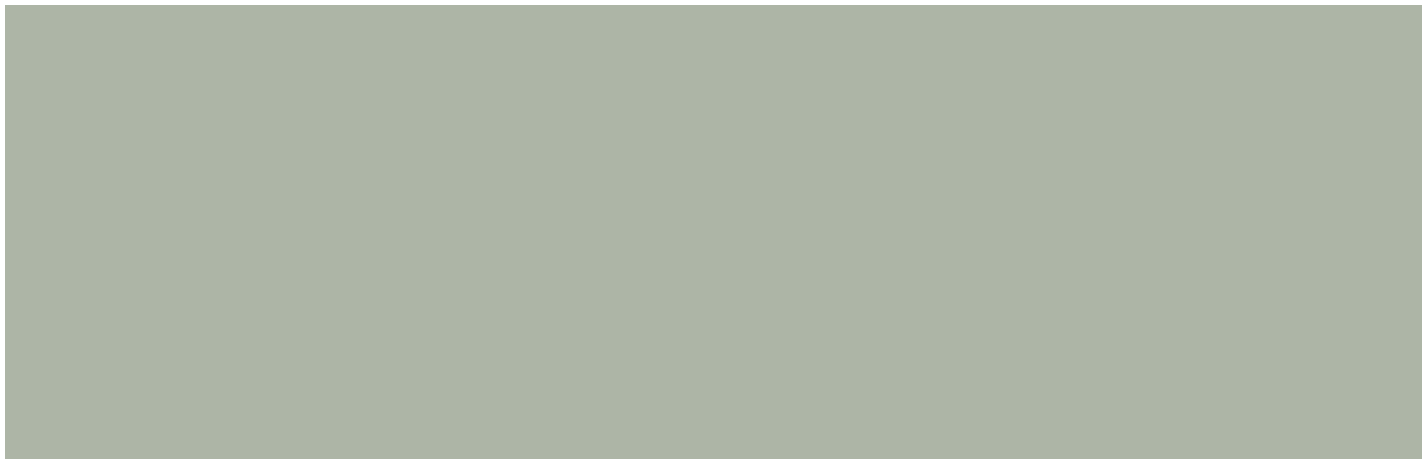

Demographics

Section A. Demographic and background information

First, we will ask you a set of questions about your personal background.

Next

0% 100%

University

1. In which university are you currently enrolled?

- Makerere University
- Mbarara University of Science and Technology (MUST)
- Rather not say

Next

0% 100%

Year

2. What year of study are you currently in?

- 4th year
- Other (please specify): Year_2_other
- Rather not say

Next

0% 100%

Gender

3. What is your gender?

- Male
- Female
- Rather not say

Next

0% 100%

Age

4. What is your age, in years?

years

Next

0% 100%

Ugandan

5. Are you currently a citizen of Uganda?

- Yes
- No (please specify country of citizenship): Ugandan_2_other
- Rather not say

Next

0% 100%

Rural

6. From the age of 5 onwards, have you at any time lived in a rural area for more than 1 year at a time (by rural we mean a settlement with a population of less than 5,000 people)?

- Yes
- No
- Rather not say

Next

0% 100%

Married

7. Are you currently married, living with a partner, or in a relationship?

- I am currently married and living with my spouse
- I am currently married but do not live with my spouse
- I am currently in a relationship and live with my partner
- I am currently in a relationship but do not live with my partner
- I am not currently in a relationship
- Rather not say

Next

0% 100%

Children

8. How many living children do you have? (please enter a figure; if none, enter 0)

children

Next

0% 100%

Religion

9. What is your religion?

- Catholic
- Anglican
- Methodist
- Presbyterian
- Other Christian
- Muslim
- Traditional/Spiritualist
- No religion
- Other (please specify): Religion_9_other
- Rather not say

Next

0% 100%

Ethnic

10. To which ethnic group or tribe do you belong?

- Baganda
- Basoga
- Iteso
- Langi
- Tutsi
- Hutu
- Bagisu
- Acholi
- Lugbara
- Banyoro
- Batooro
- Karamajong
- Other (please specify): Ethnic_13_other
- Rather not say

Next

0% 100%

Professional

Section B: Professional information

Now we are going to ask you a few questions related to your school and work experience.

Next

0% 100%

Tuition

11. How do you pay tuition for your current study program?

- I am sponsored by the government of Uganda
- I am sponsored by my home country's government (please specify the country): Tuition_2_other
- I am sponsored by a non-government organization (please specify the organization): Tuition_3_other
- I pay my tuition fee myself or with help from family and friends
- Rather not say

Next

0% 100%

Experience

12. Before enrolling in your current study program, how many years of work experience did you have as a health worker (indicate partial years with decimals; if none, enter 0)?

_____ years

Next

0% 100%

Workrural

13. Have you at any time worked as a health worker in a rural area for more than 6 months at a time? (by rural we mean a settlement with a population of less than 5,000 people)

- Yes
- No
- Rather not say

Next

0% 100%

Raterural

14. Please rate your experience overall working in a rural area?

- Excellent
- Very good
- Good
- Fair
- Poor
- Rather not say

Next

0% 100%

Bonded

15. Are you under any obligation or have you made any commitment to work in a rural area after graduation?

- Yes
- No
- Rather not say

Next

0% 100%

Schoolrural

16. During your study program thus far, have you done outreach or service in a rural area as part of your study program?

- Yes
- No
- Rather not say

Next

0% 100%

Schoolruralweeks

17. In total, how many weeks did you spend doing outreach or service in a rural area during your study program?

_____ weeks

Next

0% 100%

Ruralexperience

18. Please rate your experience overall working in a rural area during your study program?

- Excellent
- Very good
- Good
- Fair
- Poor
- Rather not say

Next

0% 100%

Likely

19. Please rate how likely you are to work in a rural area at some point in the future?

- Very unlikely
- Unlikely
- Likely
- Very likely
- Rather not say

Next

0% 100%

Mostimportant

20. Thinking in general about the possibility of rural practice, which of the following is the MOST IMPORTANT factor in your decision to work in a rural area?

- Your facility is of advanced quality (e.g. reliable electricity, equipment and drugs and supplies always available)
- You are provided basic housing
- You are allowed to own and operate 1 private retail pharmacy as long as you also work full time at the facility
- Your commitment to the position is short-term (e.g. 2 years)
- You are provided adequate salary
- The facility manager is supportive and makes work easier
- Rather not say

Next

0% 100%

Ruralwreform

21. The government of Uganda is thinking of putting in place incentives, including higher salary, free housing and better quality facilities, to encourage health workers to work in rural areas. If these incentives were available, would you consider working in a rural area?

- Yes
- No
- Rather not say

Next

0% 100%

DCE

Section C. Discrete Choice Experiment

Imagine that you have just completed your pharmacy study program. you have decided to begin working as a pharmacist in a health facility. You are checking the newspaper for available job postings, and find that there are two postings available in government run health facilities. Both of the facilities in these postings are located in rural areas. Both facilities are equal distance from the nearest big town, and are equal distance from Kampala. Also, both of these facilities are in areas that are entirely safe from violent conflict. However, each of these two postings has different benefits, including: salary, housing, the quality of the facility, the length of time you are committed, the rules regulating your ability to run a private pharmacy in addition to your work at the health facility, and support from the facility manager.

Please imagine yourself in this situation and make a real decision as to which of these two postings you would prefer. Although we know that some government benefits to health workers have not been properly implemented in the past, please assume that you will receive the full benefits described for your posting. In making your choice, please read carefully the full list of benefits for each posting and do not imagine any additional features of these postings.

Next

0% 100%

CBCRAN1

Please tell us which of these job postings you prefer.
Choose by clicking one of the buttons below:

_____	Posting A	Posting B
Quality of the facility	Basic (e.g. unreliable electricity, equipment and drugs and supplies not always available)	Advanced (e.g. reliable electricity, equipment and drugs and supplies always available)
Housing	Free basic housing provided	Housing allowance provided, enough to afford basic housing
Private opportunities	You are not allowed to own or operate any private retail pharmacies	You are allowed to own and operate 1 private retail pharmacy as long as you also work full time at the facility
Length of commitment	You are committed to this position for 5 years	You are committed to this position for 2 years
Salary	800,000 USh per month	1,200,000 USh per month
Management	The facility manager is supportive and makes work easier	The facility manager is not supportive and makes work more difficult

Next

0% 100%

CBCRAN2

Please tell us which of these job postings you prefer.
Choose by clicking one of the buttons below:

_____	Posting A	Posting B
Quality of the facility	Advanced (e.g. reliable electricity, equipment and drugs and supplies always available)	Basic (e.g. unreliable electricity, equipment and drugs and supplies not always available)
Housing	No housing or allowance provided	Free basic housing provided
Private opportunities	You are not allowed to own or operate any private retail pharmacies	You are allowed to own and operate 1 private retail pharmacy as long as you also work full time at the facility
Length of commitment	You are committed to this position for 2 years	You are committed to this position for 5 years
Salary	2,000,000 USh per month	1,600,000 USh per month
Management	The facility manager is supportive and makes work easier	The facility manager is not supportive and makes work more difficult

Next

0% 100%

CBCRAN3

Please tell us which of these job postings you prefer.
Choose by clicking one of the buttons below:

_____	Posting A	Posting B
Quality of the facility	Basic (e.g. unreliable electricity, equipment and drugs and supplies not always available)	Advanced (e.g. reliable electricity, equipment and drugs and supplies always available)
Housing	Housing allowance provided, enough to afford basic housing	No housing or allowance provided
Private opportunities	You are not allowed to own or operate any private retail pharmacies	You are allowed to own and operate 1 private retail pharmacy as long as you also work full time at the facility
Length of commitment	You are committed to this position for 2 years	You are committed to this position for 5 years
Salary	2,000,000 USh per month	1,200,000 USh per month
Management	The facility manager is not supportive and makes work more difficult	The facility manager is supportive and makes work easier

Next

0% 100%

CBCRAN4

Please tell us which of these job postings you prefer.
Choose by clicking one of the buttons below:

_____	Posting A	Posting B
Quality of the facility	Basic (e.g. unreliable electricity, equipment and drugs and supplies not always available)	Advanced (e.g. reliable electricity, equipment and drugs and supplies always available)
Housing	No housing or allowance provided	Housing allowance provided, enough to afford basic housing
Private opportunities	You are allowed to own and operate 1 private retail pharmacy as long as you also work full time at the facility	You are not allowed to own or operate any private retail pharmacies
Length of commitment	You are committed to this position for 2 years	You are committed to this position for 5 years
Salary	800,000 USh per month	1,600,000 USh per month
Management	The facility manager is supportive and makes work easier	The facility manager is not supportive and makes work more difficult

Next

0% 100%

CBCRAN5

Please tell us which of these job postings you prefer.
Choose by clicking one of the buttons below:

_____	Posting A	Posting B
Quality of the facility	Basic (e.g. unreliable electricity, equipment and drugs and supplies not always available)	Advanced (e.g. reliable electricity, equipment and drugs and supplies always available)
Housing	No housing or allowance provided	Free basic housing provided
Private opportunities	You are allowed to own and operate 1 private retail pharmacy as long as you also work full time at the facility	You are not allowed to own or operate any private retail pharmacies
Length of commitment	You are committed to this position for 5 years	You are committed to this position for 2 years
Salary	2,000,000 USh per month	1,600,000 USh per month
Management	The facility manager is not supportive and makes work more difficult	The facility manager is supportive and makes work easier

Next

0% 100%

CBCFIX1

Please tell us which of these job postings you prefer.
Choose by clicking one of the buttons below:

_____	Posting A	Posting B
Quality of the facility	Basic (e.g. unreliable electricity, equipment and drugs and supplies not always available)	Advanced (e.g. reliable electricity, equipment and drugs and supplies always available)
Housing	Free basic housing provided	Free basic housing provided
Private opportunities	You are allowed to own and operate 1 private retail pharmacy as long as you also work full time at the facility	You are not allowed to own or operate any private retail pharmacies
Length of commitment	You are committed to this position for 2 years	You are committed to this position for 5 years
Salary	1,200,000 USh per month	1,200,000 USh per month
Management	The facility manager is not supportive and makes work more difficult	The facility manager is supportive and makes work easier

Next

0% 100%

CBCRAN6

Please tell us which of these job postings you prefer.
Choose by clicking one of the buttons below:

_____	Posting A	Posting B
Quality of the facility	Advanced (e.g. reliable electricity, equipment and drugs and supplies always available)	Basic (e.g. unreliable electricity, equipment and drugs and supplies not always available)
Housing	Free basic housing provided	Housing allowance provided, enough to afford basic housing
Private opportunities	You are allowed to own and operate 1 private retail pharmacy as long as you also work full time at the facility	You are not allowed to own or operate any private retail pharmacies
Length of commitment	You are committed to this position for 2 years	You are committed to this position for 5 years
Salary	800,000 USh per month	1,200,000 USh per month
Management	The facility manager is not supportive and makes work more difficult	The facility manager is supportive and makes work easier

Next

0% 100%

CBCRAN7

Please tell us which of these job postings you prefer.
Choose by clicking one of the buttons below:

_____	Posting A	Posting B
Quality of the facility	Advanced (e.g. reliable electricity, equipment and drugs and supplies always available)	Basic (e.g. unreliable electricity, equipment and drugs and supplies not always available)
Housing	No housing or allowance provided	Housing allowance provided, enough to afford basic housing
Private opportunities	You are not allowed to own or operate any private retail pharmacies	You are allowed to own and operate 1 private retail pharmacy as long as you also work full time at the facility
Length of commitment	You are committed to this position for 5 years	You are committed to this position for 2 years
Salary	800,000 USh per month	1,600,000 USh per month
Management	The facility manager is not supportive and makes work more difficult	The facility manager is supportive and makes work easier

Next

0% 100%

CBCRAN8

Please tell us which of these job postings you prefer.
Choose by clicking one of the buttons below:

_____	Posting A	Posting B
Quality of the facility	Basic (e.g. unreliable electricity, equipment and drugs and supplies not always available)	Advanced (e.g. reliable electricity, equipment and drugs and supplies always available)
Housing	No housing or allowance provided	Free basic housing provided
Private opportunities	You are not allowed to own or operate any private retail pharmacies	You are allowed to own and operate 1 private retail pharmacy as long as you also work full time at the facility
Length of commitment	You are committed to this position for 2 years	You are committed to this position for 5 years
Salary	1,200,000 USh per month	2,000,000 USh per month
Management	The facility manager is not supportive and makes work more difficult	The facility manager is supportive and makes work easier

Next

0% 100%

CBCRAN9

Please tell us which of these job postings you prefer.
Choose by clicking one of the buttons below:

_____	Posting A	Posting B
Quality of the facility	Basic (e.g. unreliable electricity, equipment and drugs and supplies not always available)	Advanced (e.g. reliable electricity, equipment and drugs and supplies always available)
Housing	Free basic housing provided	Housing allowance provided, enough to afford basic housing
Private opportunities	You are not allowed to own or operate any private retail pharmacies	You are allowed to own and operate 1 private retail pharmacy as long as you also work full time at the facility
Length of commitment	You are committed to this position for 2 years	You are committed to this position for 5 years
Salary	1,200,000 USh per month	800,000 USh per month
Management	The facility manager is not supportive and makes work more difficult	The facility manager is supportive and makes work easier

Next

0% 100%

CBCRAN10

Please tell us which of these job postings you prefer.
Choose by clicking one of the buttons below:

_____	Posting A	Posting B
Quality of the facility	Basic (e.g. unreliable electricity, equipment and drugs and supplies not always available)	Advanced (e.g. reliable electricity, equipment and drugs and supplies always available)
Housing	No housing or allowance provided	Free basic housing provided
Private opportunities	You are not allowed to own or operate any private retail pharmacies	You are allowed to own and operate 1 private retail pharmacy as long as you also work full time at the facility
Length of commitment	You are committed to this position for 5 years	You are committed to this position for 2 years
Salary	1,600,000 USh per month	2,000,000 USh per month
Management	The facility manager is supportive and makes work easier	The facility manager is not supportive and makes work more difficult

Next

0% 100%

CBCRAN11

Please tell us which of these job postings you prefer.
Choose by clicking one of the buttons below:

_____	Posting A	Posting B
Quality of the facility	Basic (e.g. unreliable electricity, equipment and drugs and supplies not always available)	Advanced (e.g. reliable electricity, equipment and drugs and supplies always available)
Housing	Free basic housing provided	Housing allowance provided, enough to afford basic housing
Private opportunities	You are allowed to own and operate 1 private retail pharmacy as long as you also work full time at the facility	You are not allowed to own or operate any private retail pharmacies
Length of commitment	You are committed to this position for 5 years	You are committed to this position for 2 years
Salary	1,200,000 USh per month	800,000 USh per month
Management	The facility manager is not supportive and makes work more difficult	The facility manager is supportive and makes work easier

Next

0% 100%

Comments

Do you have any other comments or feedback pertaining to this survey?

Next

0% 100%

Thanks

Thank you very much for your participation.

Raise your hand to notify the facilitator that you have finished.

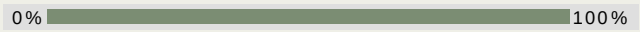

Nonconsent

Thank you very much.

Raise your hand to notify the facilitator that you have finished.

0% 100%
